# (Benzyl phenyl sulfoxide-κ*O*)dichloridodiphenyl­tin(IV)

**DOI:** 10.1107/S1600536811014474

**Published:** 2011-04-29

**Authors:** Guo-Xia Tan, Chang-Fa Zhang, Xi-Cheng Liu

**Affiliations:** a Experimental Center, Linyi University, Linyi 276000, People’s Republic of China; b Shandong Water Polytechnic, Rizhao 276826, People’s Republic of China; c Department of Chemistry, Qufu Normal University, Qufu 273165, People’s Republic of China

## Abstract

The Sn^IV^ atom in the title compound, [Sn(C_6_H_5_)_2_Cl_2_(C_13_H_12_OS)], displays a distorted C_2_Cl_2_O trigonal–bipyramidal coordination environment, with a mean Sn—C distance of 2.121 (9) Å and with Sn—O = 2.331 (2) Å. The Sn^IV^ atom is displaced by 0.169 (2) Å from the equatorial C_2_Cl plane towards the direction of the second axially bonded Cl atom.

## Related literature

For background to organotin compounds, see: Davies *et al.* (2008 ▶); Tian *et al.* (2005 ▶). For related structures, see: Sadiq-ur-Rehman *et al.* (2007 ▶); Ng & Rheingold (1989 ▶); Bao *et al.* (1995 ▶); Dang (2009 ▶); Sousa *et al.* (2009 ▶); Yu *et al.* (1992 ▶).
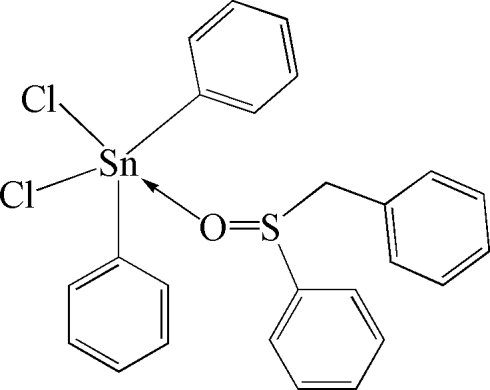


## Experimental

### 

#### Crystal data

[Sn(C_6_H_5_)_2_Cl_2_(C_13_H_12_OS)]
*M* *_r_* = 560.08
Triclinic, 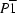

*a* = 9.979 (5) Å
*b* = 10.577 (6) Å
*c* = 12.104 (4) Å
α = 87.728 (5)°
β = 81.196 (3)°
γ = 69.995 (5)°
*V* = 1186.2 (10) Å^3^
*Z* = 2
Mo *K*α radiation
μ = 1.41 mm^−1^
*T* = 295 K
0.31 × 0.24 × 0.20 mm

#### Data collection

Bruker *P*4 diffractometer
Absorption correction: ψ scan (*XSCANS*; Bruker, 1996 ▶) *T*_min_ = 0.670, *T*_max_ = 0.766
4959 measured reflections
4170 independent reflections
3546 reflections with *I* > 2σ(*I*)
*R*_int_ = 0.020
3 standard reflections every 97 reflections  intensity decay: 1.9%

#### Refinement

*R*[*F* ^2^ > 2σ(*F* ^2^)] = 0.025
*wR*(*F* ^2^) = 0.056
*S* = 1.01
4170 reflections
272 parameters
H-atom parameters constrained
Δρ_max_ = 0.26 e Å^−3^
Δρ_min_ = −0.34 e Å^−3^

### 

Data collection: *XSCANS* (Bruker, 1996 ▶); cell refinement: *XSCANS*; data reduction: *XSCANS*; program(s) used to solve structure: *SHELXS97* (Sheldrick, 2008 ▶); program(s) used to refine structure: *SHELXL97* (Sheldrick, 2008 ▶); molecular graphics: *ORTEP-3 for Windows* (Farrugia, 1997 ▶); software used to prepare material for publication: *publCIF* (Westrip, 2010 ▶).

## Supplementary Material

Crystal structure: contains datablocks global, I. DOI: 10.1107/S1600536811014474/wm2481sup1.cif

Structure factors: contains datablocks I. DOI: 10.1107/S1600536811014474/wm2481Isup2.hkl

Additional supplementary materials:  crystallographic information; 3D view; checkCIF report

## Figures and Tables

**Fig. 1.**
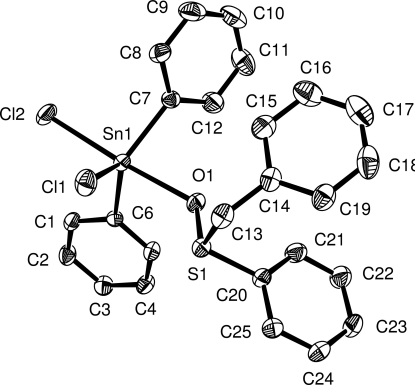
The molecular structure of (I) with displacement ellipsoids drawn at the 30% probability level.

## supplementary crystallographic information

### Comment

Organotin compounds have received considerable attention due to their structural diversity and an increase in terms of industrial, agricultural and biological applications (Davies *et al.*, 2008; Tian *et al.*, 2005).

Several structures of organotin sulfoxide complexes, such as dichloridobis(dimethylsulfoxide-κ*O*)diphenyltin (Sadiq-ur-Rehman *et al.*, 2007), dichloridodimethyl(dibenzylsulfoxide-κ*O*)tin (Ng & Rheingold, 1989), [bis(phenylsulfinyl)ethane-κ*O*,*O*']dichloridodiphenyltin (Bao *et al.*, 1995), bis(benzylphenylsulfoxide-κ*O*)dichloridodiphenyltin (Dang, 2009) or dibutyldichloro(2-phenyl-1,3-dithiane-1,3-dioxide-κ*O*)tin (Sousa *et al.*, 2009), have been reported. As a continuation of these studies, the structure of the title compound, (I), is described here.

The coordination environment of the Sn^IV^ atom in (I) can be described as a distorted trigonal bipyramid with two phenyl groups and the Cl1 atom occupying the equatorial positions whereas the axial positions are occupied by the Cl2 atom and the sulfoxide O1 atom (Fig. 1). The Sn atom is slightly displaced from the equatorial plane defined by the C_2_Cl set and is located 0.169 (2) Å in the direction of the axial Cl2 atom. The Sn—C and Sn—Cl bond lengths are similar to these found in [1,2-bis(phenylsulfinyl)ethane-κ*O*,*O*]bis(dichloridodiphenyltin) (Yu *et al.*, 1992). However, the Sn—O bond length (2.331 (2) Å) is shorter than that in the previously mentioned structure (2.417 (2) Å). The dihedral angle between the two phenyl rings bound to the Sn atom is 55.9 (2)°; the dihedral angle between the phenyl rings in the sulfoxide ligand is 34.7 (2)°.

### Experimental

Benzylphenylsulfoxide (0.865 g, 4 mmol) and diphenyltin dichloride (1.374 g, 4 mmol) were refluxed in methanol (40 ml) for 1 h, and then the colorless solution was concentrated under reduce pressure and cooled. The solid product obtained was filtered off and recrystallised from ethanol. Colourless crystals suitable for X-ray analysis were obtained from the solvent by slow evaporation (yield 80%; m.p. 378–379 K).

### Refinement

H atoms were placed at calculated positions (C—H = 0.97 Å for methylene and C—H = 0.93 Å for aromatic H atoms) and refined as riding with *U*_iso_(H) = 1.2*U*_eq_(C).

### Crystal data

**Table:**

[Sn(C_6_H_5_)_2_Cl_2_(C_13_H_12_OS)]	*Z* = 2
*M**_r_* = 560.08	*F*(000) = 560
Triclinic, *P*1	*D*_x_ = 1.568 Mg m^−^^3^
Hall symbol: -P 1	Mo *K*α radiation, λ = 0.71069 Å
*a* = 9.979 (5) Å	Cell parameters from 28 reflections
*b* = 10.577 (6) Å	θ = 5.4–12.5°
*c* = 12.104 (4) Å	µ = 1.41 mm^−^^1^
α = 87.728 (5)°	*T* = 295 K
β = 81.196 (3)°	Block, colourless
γ = 69.995 (5)°	0.31 × 0.24 × 0.20 mm
*V* = 1186.2 (10) Å^3^	

### Data collection

**Table:**

Bruker P4 diffractometer	3546 reflections with *I* > 2σ(*I*)
Radiation source: fine-focus sealed tube	*R*_int_ = 0.020
graphite	θ_max_ = 25.0°, θ_min_ = 2.1°
ω scans	*h* = −11→1
Absorption correction: ψ scan (*XSCANS*; Bruker, 1996)	*k* = −12→12
*T*_min_ = 0.670, *T*_max_ = 0.766	*l* = −14→14
4959 measured reflections	3 standard reflections every 97 reflections
4170 independent reflections	intensity decay: 1.9%

### Refinement

**Table:**

Refinement on *F*^2^	Secondary atom site location: difference Fourier map
Least-squares matrix: full	Hydrogen site location: inferred from neighbouring sites
*R*[*F*^2^ > 2σ(*F*^2^)] = 0.025	H-atom parameters constrained
*wR*(*F*^2^) = 0.056	*w* = 1/[σ^2^(*F*_o_^2^) + (0.0246*P*)^2^ + 0.1469*P*] where *P* = (*F*_o_^2^ + 2*F*_c_^2^)/3
*S* = 1.01	(Δ/σ)_max_ = 0.001
4170 reflections	Δρ_max_ = 0.26 e Å^−^^3^
272 parameters	Δρ_min_ = −0.34 e Å^−^^3^
0 restraints	Extinction correction: *SHELXL97* (Sheldrick, 2008), Fc^*^=kFc[1+0.001xFc^2^λ^3^/sin(2θ)]^-1/4^
Primary atom site location: structure-invariant direct methods	Extinction coefficient: 0.0111 (5)

### Special details

**Table:**

Geometry. All e.s.d.'s (except the e.s.d. in the dihedral angle between two l.s. planes) are estimated using the full covariance matrix. The cell e.s.d.'s are taken into account individually in the estimation of e.s.d.'s in distances, angles and torsion angles; correlations between e.s.d.'s in cell parameters are only used when they are defined by crystal symmetry. An approximate (isotropic) treatment of cell e.s.d.'s is used for estimating e.s.d.'s involving l.s. planes.
Refinement. Refinement of *F*^2^ against ALL reflections. The weighted *R*-factor *wR* and goodness of fit *S* are based on *F*^2^, conventional *R*-factors *R* are based on *F*, with *F* set to zero for negative *F*^2^. The threshold expression of *F*^2^ > σ(*F*^2^) is used only for calculating *R*-factors(gt) *etc*. and is not relevant to the choice of reflections for refinement. *R*-factors based on *F*^2^ are statistically about twice as large as those based on *F*, and *R*-factors based on ALL data will be even larger.

### Fractional atomic coordinates and isotropic or equivalent isotropic displacement parameters (Å^2^)

**Table:**

	*x*	*y*	*z*	*U*_iso_*/*U*_eq_	
C1	0.7386 (3)	0.1721 (3)	1.0500 (2)	0.0430 (7)	
H1A	0.8239	0.1008	1.0298	0.052*	
C2	0.7161 (3)	0.2382 (3)	1.1502 (3)	0.0522 (8)	
H2A	0.7858	0.2109	1.1974	0.063*	
C3	0.5907 (3)	0.3446 (3)	1.1814 (3)	0.0495 (8)	
H3A	0.5759	0.3890	1.2493	0.059*	
C4	0.4878 (3)	0.3847 (3)	1.1118 (3)	0.0503 (8)	
H4A	0.4032	0.4567	1.1325	0.060*	
C5	0.5097 (3)	0.3183 (3)	1.0111 (2)	0.0442 (7)	
H5A	0.4394	0.3459	0.9644	0.053*	
C6	0.6355 (3)	0.2108 (3)	0.9784 (2)	0.0358 (6)	
C7	0.7105 (3)	0.1817 (3)	0.6623 (2)	0.0398 (6)	
C8	0.7903 (3)	0.0972 (3)	0.5733 (3)	0.0545 (8)	
H8A	0.8277	0.0049	0.5841	0.065*	
C9	0.8148 (4)	0.1490 (5)	0.4686 (3)	0.0718 (11)	
H9A	0.8674	0.0916	0.4091	0.086*	
C10	0.7618 (4)	0.2846 (5)	0.4527 (3)	0.0731 (11)	
H10A	0.7797	0.3194	0.3824	0.088*	
C11	0.6830 (5)	0.3691 (4)	0.5390 (3)	0.0814 (12)	
H11A	0.6472	0.4614	0.5277	0.098*	
C12	0.6561 (4)	0.3176 (3)	0.6440 (3)	0.0619 (9)	
H12A	0.6007	0.3755	0.7025	0.074*	
C13	0.2765 (3)	0.0805 (3)	0.7273 (3)	0.0526 (8)	
H13A	0.3563	−0.0034	0.7113	0.063*	
H13B	0.1895	0.0584	0.7488	0.063*	
C14	0.2623 (3)	0.1608 (3)	0.6234 (3)	0.0473 (7)	
C15	0.3841 (4)	0.1643 (4)	0.5531 (3)	0.0602 (9)	
H15A	0.4750	0.1160	0.5710	0.072*	
C16	0.3713 (5)	0.2394 (4)	0.4561 (3)	0.0794 (12)	
H16A	0.4535	0.2421	0.4095	0.095*	
C17	0.2376 (6)	0.3093 (4)	0.4293 (4)	0.0875 (13)	
H17A	0.2291	0.3590	0.3640	0.105*	
C18	0.1176 (5)	0.3065 (4)	0.4974 (4)	0.0804 (12)	
H18A	0.0272	0.3550	0.4788	0.096*	
C19	0.1282 (4)	0.2322 (3)	0.5946 (3)	0.0607 (9)	
H19A	0.0451	0.2303	0.6404	0.073*	
C20	0.1536 (3)	0.3141 (3)	0.8516 (2)	0.0427 (7)	
C21	0.1612 (3)	0.4285 (3)	0.7968 (3)	0.0536 (8)	
H21A	0.2477	0.4298	0.7561	0.064*	
C22	0.0383 (4)	0.5415 (3)	0.8031 (3)	0.0615 (9)	
H22A	0.0424	0.6201	0.7677	0.074*	
C23	−0.0899 (4)	0.5380 (3)	0.8615 (3)	0.0599 (9)	
H23A	−0.1727	0.6136	0.8648	0.072*	
C24	−0.0951 (3)	0.4224 (4)	0.9152 (3)	0.0598 (9)	
H24A	−0.1821	0.4206	0.9545	0.072*	
C25	0.0255 (3)	0.3099 (3)	0.9117 (3)	0.0537 (8)	
H25A	0.0215	0.2323	0.9489	0.064*	
Cl1	0.61881 (10)	−0.09242 (8)	0.83415 (8)	0.0626 (2)	
Cl2	0.93145 (8)	−0.02330 (10)	0.82750 (7)	0.0661 (3)	
O1	0.43433 (19)	0.20989 (19)	0.80186 (16)	0.0443 (5)	
S1	0.30677 (8)	0.16446 (8)	0.84580 (6)	0.04423 (19)	
Sn1	0.67532 (2)	0.10548 (2)	0.824114 (16)	0.03808 (9)	

### Atomic displacement parameters (Å^2^)

**Table:**

	*U*^11^	*U*^22^	*U*^33^	*U*^12^	*U*^13^	*U*^23^
C1	0.0342 (16)	0.0413 (16)	0.0478 (17)	−0.0049 (13)	−0.0073 (13)	0.0003 (13)
C2	0.0475 (19)	0.0552 (19)	0.0500 (18)	−0.0069 (16)	−0.0201 (15)	−0.0006 (15)
C3	0.054 (2)	0.0461 (18)	0.0476 (18)	−0.0138 (16)	−0.0096 (15)	−0.0060 (14)
C4	0.0411 (18)	0.0449 (18)	0.0565 (19)	−0.0040 (14)	−0.0056 (15)	−0.0063 (14)
C5	0.0358 (16)	0.0427 (17)	0.0503 (17)	−0.0068 (13)	−0.0109 (14)	0.0014 (13)
C6	0.0322 (15)	0.0379 (15)	0.0371 (14)	−0.0128 (12)	−0.0038 (12)	0.0041 (12)
C7	0.0388 (16)	0.0450 (16)	0.0362 (15)	−0.0150 (13)	−0.0053 (12)	−0.0022 (12)
C8	0.0489 (19)	0.057 (2)	0.0492 (19)	−0.0089 (16)	−0.0008 (15)	−0.0081 (15)
C9	0.069 (3)	0.104 (3)	0.0415 (19)	−0.030 (2)	0.0020 (18)	−0.014 (2)
C10	0.082 (3)	0.110 (3)	0.045 (2)	−0.056 (3)	−0.0114 (19)	0.017 (2)
C11	0.115 (4)	0.062 (2)	0.075 (3)	−0.038 (2)	−0.024 (3)	0.025 (2)
C12	0.075 (2)	0.050 (2)	0.053 (2)	−0.0143 (18)	−0.0022 (18)	−0.0014 (16)
C13	0.0453 (18)	0.0452 (18)	0.070 (2)	−0.0173 (15)	−0.0125 (16)	−0.0030 (15)
C14	0.0482 (19)	0.0409 (17)	0.0559 (19)	−0.0146 (15)	−0.0163 (15)	−0.0074 (14)
C15	0.053 (2)	0.066 (2)	0.061 (2)	−0.0161 (18)	−0.0111 (17)	−0.0123 (18)
C16	0.093 (3)	0.097 (3)	0.058 (2)	−0.046 (3)	−0.010 (2)	−0.001 (2)
C17	0.123 (4)	0.090 (3)	0.064 (3)	−0.048 (3)	−0.035 (3)	0.013 (2)
C18	0.086 (3)	0.072 (3)	0.086 (3)	−0.014 (2)	−0.048 (3)	0.000 (2)
C19	0.051 (2)	0.064 (2)	0.070 (2)	−0.0184 (18)	−0.0192 (18)	−0.0073 (18)
C20	0.0330 (16)	0.0493 (17)	0.0436 (16)	−0.0112 (14)	−0.0061 (13)	0.0009 (13)
C21	0.0438 (18)	0.054 (2)	0.061 (2)	−0.0180 (16)	0.0014 (15)	0.0038 (16)
C22	0.055 (2)	0.049 (2)	0.073 (2)	−0.0116 (17)	−0.0039 (18)	0.0097 (17)
C23	0.0437 (19)	0.053 (2)	0.070 (2)	−0.0004 (16)	−0.0088 (17)	−0.0004 (17)
C24	0.0327 (17)	0.073 (2)	0.068 (2)	−0.0128 (17)	−0.0013 (15)	0.0051 (18)
C25	0.0412 (18)	0.061 (2)	0.0567 (19)	−0.0175 (16)	−0.0034 (15)	0.0114 (16)
Cl1	0.0650 (6)	0.0415 (4)	0.0815 (6)	−0.0173 (4)	−0.0152 (5)	0.0066 (4)
Cl2	0.0340 (4)	0.0797 (6)	0.0649 (5)	0.0063 (4)	−0.0071 (4)	−0.0047 (4)
O1	0.0304 (10)	0.0503 (12)	0.0515 (12)	−0.0122 (9)	−0.0082 (9)	0.0028 (9)
S1	0.0355 (4)	0.0484 (4)	0.0464 (4)	−0.0117 (3)	−0.0062 (3)	0.0047 (3)
Sn1	0.03092 (12)	0.04049 (12)	0.03830 (12)	−0.00709 (8)	−0.00347 (8)	0.00064 (8)

### Geometric parameters (Å, °)

**Table:**

C1—C2	1.373 (4)	C14—C19	1.383 (4)
C1—C6	1.391 (4)	C14—C15	1.383 (4)
C1—H1A	0.9300	C15—C16	1.389 (5)
C2—C3	1.378 (4)	C15—H15A	0.9300
C2—H2A	0.9300	C16—C17	1.368 (6)
C3—C4	1.372 (4)	C16—H16A	0.9300
C3—H3A	0.9300	C17—C18	1.355 (6)
C4—C5	1.382 (4)	C17—H17A	0.9300
C4—H4A	0.9300	C18—C19	1.386 (5)
C5—C6	1.390 (4)	C18—H18A	0.9300
C5—H5A	0.9300	C19—H19A	0.9300
C6—Sn1	2.127 (3)	C20—C21	1.376 (4)
C7—C12	1.373 (4)	C20—C25	1.385 (4)
C7—C8	1.386 (4)	C20—S1	1.780 (3)
C7—Sn1	2.114 (3)	C21—C22	1.384 (4)
C8—C9	1.380 (5)	C21—H21A	0.9300
C8—H8A	0.9300	C22—C23	1.376 (4)
C9—C10	1.365 (5)	C22—H22A	0.9300
C9—H9A	0.9300	C23—C24	1.375 (5)
C10—C11	1.360 (5)	C23—H23A	0.9300
C10—H10A	0.9300	C24—C25	1.370 (4)
C11—C12	1.387 (5)	C24—H24A	0.9300
C11—H11A	0.9300	C25—H25A	0.9300
C12—H12A	0.9300	Cl1—Sn1	2.3410 (13)
C13—C14	1.487 (4)	Cl2—Sn1	2.4567 (14)
C13—S1	1.831 (3)	O1—S1	1.525 (2)
C13—H13A	0.9700	O1—Sn1	2.331 (2)
C13—H13B	0.9700		
			
C2—C1—C6	120.8 (3)	C14—C15—H15A	119.8
C2—C1—H1A	119.6	C16—C15—H15A	119.8
C6—C1—H1A	119.6	C17—C16—C15	119.8 (4)
C1—C2—C3	120.4 (3)	C17—C16—H16A	120.1
C1—C2—H2A	119.8	C15—C16—H16A	120.1
C3—C2—H2A	119.8	C18—C17—C16	120.3 (4)
C4—C3—C2	119.8 (3)	C18—C17—H17A	119.9
C4—C3—H3A	120.1	C16—C17—H17A	119.9
C2—C3—H3A	120.1	C17—C18—C19	120.7 (4)
C3—C4—C5	120.0 (3)	C17—C18—H18A	119.6
C3—C4—H4A	120.0	C19—C18—H18A	119.6
C5—C4—H4A	120.0	C14—C19—C18	120.0 (4)
C4—C5—C6	120.9 (3)	C14—C19—H19A	120.0
C4—C5—H5A	119.5	C18—C19—H19A	120.0
C6—C5—H5A	119.5	C21—C20—C25	121.3 (3)
C5—C6—C1	118.1 (3)	C21—C20—S1	121.5 (2)
C5—C6—Sn1	122.5 (2)	C25—C20—S1	117.2 (2)
C1—C6—Sn1	119.4 (2)	C20—C21—C22	119.0 (3)
C12—C7—C8	118.6 (3)	C20—C21—H21A	120.5
C12—C7—Sn1	120.1 (2)	C22—C21—H21A	120.5
C8—C7—Sn1	121.3 (2)	C23—C22—C21	120.2 (3)
C9—C8—C7	120.5 (3)	C23—C22—H22A	119.9
C9—C8—H8A	119.8	C21—C22—H22A	119.9
C7—C8—H8A	119.8	C24—C23—C22	119.8 (3)
C10—C9—C8	119.9 (3)	C24—C23—H23A	120.1
C10—C9—H9A	120.0	C22—C23—H23A	120.1
C8—C9—H9A	120.0	C25—C24—C23	121.2 (3)
C11—C10—C9	120.4 (3)	C25—C24—H24A	119.4
C11—C10—H10A	119.8	C23—C24—H24A	119.4
C9—C10—H10A	119.8	C24—C25—C20	118.5 (3)
C10—C11—C12	120.0 (4)	C24—C25—H25A	120.7
C10—C11—H11A	120.0	C20—C25—H25A	120.7
C12—C11—H11A	120.0	S1—O1—Sn1	127.97 (11)
C7—C12—C11	120.5 (3)	O1—S1—C20	104.46 (13)
C7—C12—H12A	119.7	O1—S1—C13	105.54 (13)
C11—C12—H12A	119.7	C20—S1—C13	98.91 (14)
C14—C13—S1	114.6 (2)	C7—Sn1—C6	126.64 (11)
C14—C13—H13A	108.6	C7—Sn1—O1	82.35 (9)
S1—C13—H13A	108.6	C6—Sn1—O1	89.84 (9)
C14—C13—H13B	108.6	C7—Sn1—Cl1	116.24 (8)
S1—C13—H13B	108.6	C6—Sn1—Cl1	115.33 (8)
H13A—C13—H13B	107.6	O1—Sn1—Cl1	84.46 (6)
C19—C14—C15	118.8 (3)	C7—Sn1—Cl2	94.39 (8)
C19—C14—C13	121.0 (3)	C6—Sn1—Cl2	97.34 (8)
C15—C14—C13	120.2 (3)	O1—Sn1—Cl2	172.69 (5)
C14—C15—C16	120.4 (3)	Cl1—Sn1—Cl2	91.18 (4)

## References

[bb1] Bao, J. C., Shao, P. X., Wang, R. J., Wang, H. G. & Yao, X. K. (1995). *Polyhedron*, **14**, 927–933.

[bb2] Bruker (1996). *XSCANS* Bruker AXS Inc., Madison, Wisconsin, USA.

[bb3] Dang, Y.-Q. (2009). *Acta Cryst.* E**65**, m1306. 10.1107/S1600536809039269 PMC2971230 21578070

[bb4] Davies, A. G., Gielen, M., Pannell, K. H. & Tiekink, E. R. T. (2008). In *Tin Chemistry: Fundamentals, Frontiers and Applications* Chichester: John Wiley & Sons.

[bb5] Farrugia, L. J. (1997). *J. Appl. Cryst.* **30**, 565.

[bb6] Ng, S. W. & Rheingold, A. L. (1989). *J. Organomet. Chem.* **378**, 339–345.

[bb7] Sadiq-ur-Rehman, Saeed, S., Ali, S., Shahzadi, S. & Helliwell, M. (2007). *Acta Cryst.* E**63**, m1788.

[bb8] Sheldrick, G. M. (2008). *Acta Cryst.* A**64**, 112–122. 10.1107/S0108767307043930 18156677

[bb9] Sousa, G. F., Ellena, J., Malta, V. R. S. & Ardisson, J. D. (2009). *J. Braz. Chem. Soc.* **20**, 1441–1447.

[bb10] Tian, L., Sun, Y., Li, H., Zheng, X., Cheng, Y., Liu, X. & Qian, B. (2005). *J. Inorg. Biochem.* **99**, 1646–1652. 10.1016/j.jinorgbio.2005.05.006 15967504

[bb11] Westrip, S. P. (2010). *J. Appl. Cryst.* **43**, 920–925.

[bb12] Yu, X.-L., Bao, J.-C., Shao, P.-X., Yao, X.-K., Wang, H.-G. & Wang, R.-J. (1992). *Chin. J. Struct. Chem.* **11**, 373–376.

